# Validation of the Equine Urticaria Activity Score for the assessment of chronic recurrent urticaria in horses

**DOI:** 10.1111/vde.13358

**Published:** 2025-05-19

**Authors:** Katharina Birkmann, Nina Waldern, Simone Jucker, Katharina Balaschitsch, Yury Zablotski, Antonia Fettelschoss‐Gabriel

**Affiliations:** ^1^ Evax AG Guntershausen Switzerland; ^2^ Equine Clinic Ludwig‐Maximilians University Munich Oberschleißheim Germany; ^3^ Clinic for Ruminants with Ambulatory and Herd Health Services Ludwig‐Maximilians University Munich Oberschleißheim Germany; ^4^ Department of Dermatology University Hospital Zurich Zürich Switzerland; ^5^ Faculty of Medicine University of Zurich Zürich Switzerland

**Keywords:** chronic recurrent urticaria, Equine Urticaria Activity Score), grading system, horse, reliability

## Abstract

**Background:**

Recurrent urticaria is common in horses. The pathophysiology is poorly understood and treatment options are limited. Often, only glucocorticoids are effective for controlling clinical signs, albeit with potential adverse effects. Studies investigating new treatments need a validated objective scoring system for the grading of skin lesions to assess response.

**Hypothesis/Objectives:**

The aims were to investigate inter‐ and intraobserver reliability of the Equine Urticaria Activity Score (EqUAS) for the grading of skin lesions in horses with recurrent urticaria, and to examine agreement between experienced and inexperienced observers.

**Animals:**

Forty privately owned horses enrolled in a therapeutic clinical trial.

**Materials and Methods:**

Ten standardised photographs of both sides of the body of each horse were used to create 40 individual datasets. Five masked observers graded the photographs according to the EqUAS for calculation of interobserver reliability. Intraobserver reliability was calculated from two observers each grading the photographs twice. Scores were evaluated for reliability with Pearson's correlation (*r*) and for agreement with the intraclass correlation coefficient (ICC).

**Results:**

Interobserver reliability was excellent with *r* = 0.9036–0.9600 (*p* < 0.0001). Intraobserver reliability and agreement also were excellent with *r* = 0.98 for each observer (*p* < 0.0001) and ICC = 0.945. Correlation and agreement between experienced and inexperienced observers were excellent (*r* = 0.9615 [*p* < 0.0001] and ICC = 0.941).

**Conclusions and Clinical Relevance:**

The EqUAS showed excellent intra‐ and interobserver reliability for the evaluation of skin lesions of horses with chronic recurrent urticaria. It can be a useful tool in clinical studies.

## Graphical abstract

**Background** – Recurrent urticaria is common in horses. The pathophysiology is poorly understood and treatment options are limited. Often only glucocorticoids are effective for controlling clinical signs, albeit with potential adverse effects. Studies investigating new treatments need a validated objective scoring system for the grading of skin lesions to assess response. **Hypothesis/Objectives** – The aims were to investigate inter‐ and intraobserver reliability of the Equine Urticaria Activity Score (EqUAS) for the grading of skin lesions in horses with recurrent urticaria, and to examine agreement between experienced and inexperienced observers. **Conclusions and Clinical Relevance** – The EqUAS showed excellent intra‐ and interobserver reliability for the evaluation of skin lesions of horses with chronic recurrent urticaria. It can be a useful tool in clinical studies.

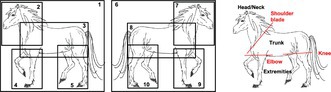

## Zusammenfassung

**Hintergrund:**

Wiederkehrende Urtikaria treten bei Pferden häufig auf. Die Pathophysiologie ist noch wenig bekannt und die Behandlungsoptionen sind limitiert. Oft sind nur Glukokortikoide zur Kontrolle der klinischen Zeichen wirksam, obwohl sie mögliche Nebenwirkungen haben. Studien, die neue Behandlungen untersuchen, benötigen ein validiertes objektives Bewertungssystem, um die Hautläsionen zu bewerten und eine Verbesserung beurteilen zu können.

**Hypothese/Ziele:**

Die Ziele dieser Studie waren die Untersuchung der Inter‐ und Intraobserver Reliabilität des Equine Urticaria Activity Score (EqUAS) zur Bewertung von Hautläsionen bei Pferden mit wiederkehrender Urtikaria und eine Untersuchung der Übereinstimmung zwischen erfahrenen und unerfahrenen BeobachterInnen.

**Tiere:**

40 Pferde in Privatbesitz wurden in diese therapeutische klinische Studie aufgenommen.

**Materialien und Methoden:**

Zehn standardisierte Fotografien von beiden Seiten des Körpers eines jeden Pferdes wurden eingesetzt, um 40 individuelle Datensets zu schaffen. Fünf geblindete BeobachterInnen beurteilten die Fotografien entsprechend dem EqUAS, um die Interobserver Reliabilität zu kalkulieren. Die Intraobserver Reliabilität wurde aus zwei BeobachterInnen kalkuliert, wobei jedes Foto zweimal beurteilt wurde.

**Ergebnisse:**

Die Interobserver Reliabilität war exzellent mit r = 0,9036‐0,9600 (p < 0,0001). Die Intraobserver Reliabilität und diese Übereinstimmung war ebenfalls exzellent mit r = 0,98 für jede/n BeobachterIn (p < 0,0001) und ICC = 0,945. Die Korrelation und die Übereinstimmung zwischen erfahrenen und unerfahrenen BeobachterInnen war exzellent (r = 0,9615 [p < 0,0001] und ICC = 0,941).

**Schlussfolgerungen und klinische Bedeutung:**

Die EqUAS zeigte exzellente Intra‐ und Interobserver Reliabilität zur Evaluierung von Hautläsionen bei Pferden mit chronisch wiederkehrender Urtikaria. Es kann bei klinischen Studien ein wichtiges Instrument sein.

## 摘要

**背景:**

复发性荨麻疹在马中很常见。其病理生理机制尚不清楚，治疗方案也有限。通常只有糖皮质激素才能有效控制临床症状，尽管存在潜在的副作用。研究新疗法需要一种经过验证的客观评分系统来对皮肤病变进行分级，以评估疗效。

**假设/目标:**

旨在探讨马荨麻疹活动评分 (EqUAS) 对复发性荨麻疹马皮肤病变进行分级的观察者间和观察者内信度，并检验经验丰富和缺乏经验的观察者之间的一致性。

**动物:**

40 匹参与治疗性临床试验的私人饲养马。

**材料与方法:**

使用每匹马身体两侧的十张标准化照片，创建 40 个单独的数据集。五名设盲观察员根据 EqUAS 对照片进行分级，以计算观察者间信度。观察者内信度由两位观察者计算得出，每位观察者对照片进行两次评分。使用皮尔逊相关系数 (r) 评估信度，并使用组内相关系数 (ICC) 评估一致性。

**结果:**

观察者间信度极佳，r = 0.9036–0.9600(p < 0.0001)。观察者内信度和一致性也极佳，每位观察者的 r = 0.98(p < 0.0001)，ICC = 0.945。经验丰富和经验不足的观察者之间的相关性和一致性极佳(r = 0.9615 [p < 0.0001])，ICC = 0.941)。

**结论与临床意义:**

EqUAS 在评估患有慢性复发性荨麻疹的马的皮肤病变方面表现出极佳的观察者内和观察者间信度。它可以成为临床研究中的有用工具。

## Résumé

**Contexte:**

L'urticaire récurrente est fréquente chez les chevaux. La physiopathologie est mal comprise et les options thérapeutiques sont limitées. Souvent, seuls les glucocorticoïdes sont efficaces pour contrôler les signes cliniques, bien qu'ils aient des effets indésirables potentiels. Les études portant sur de nouveaux traitements ont besoin d'un système de notation objectif et validé pour classer les lésions cutanées afin d'évaluer la réponse.

**Hypothèse/objectifs:**

Les objectifs étaient d'étudier la fiabilité inter‐ et intra‐observateur de l'Equine Urticaria Activity Score (EqUAS) pour le classement des lésions cutanées chez les chevaux souffrant d'urticaire récurrente, et d'examiner l'accord entre les observateurs expérimentés et inexpérimentés.

**Animaux:**

40 chevaux privés participant à un essai clinique thérapeutique.

**Matériels et méthodes:**

Dix photographies standardisées des deux côtés du corps de chaque cheval ont été utilisées pour créer 40 ensembles de données individuels. Cinq observateurs masqués ont noté les photographies selon l'EqUAS pour le calcul de la fiabilité interobservateurs. La fiabilité intra‐observateur a été calculée à partir de deux observateurs notant chacun deux fois les photographies. Les scores ont été évalués pour la fiabilité avec la corrélation de Pearson (r) et pour la concordance avec le coefficient de corrélation intraclasse (ICC).

**Résultats:**

La fiabilité interobservateurs était excellente avec r = 0,9036‐0,9600 (p < 0,0001). La fiabilité et la concordance intra‐observateur étaient également excellentes avec r = 0,98 pour chaque observateur (p < 0,0001) et ICC = 0,945. La corrélation et l'accord entre les observateurs expérimentés et inexpérimentés étaient excellents (r = 0,9615 [p < 0,0001]) et ICC = 0,941).

**Conclusions et pertinence clinique:**

L'EqUAS a montré une excellente fiabilité intra‐ et inter‐observateurs pour l'évaluation des lésions cutanées des chevaux atteints d'urticaire chronique récurrente. Il peut être un outil utile dans les études cliniques.

## 要約

**背景:**

再発性蕁麻疹は馬によくみられる疾患である。その病態生理学は十分に理解されておらず、治療選択肢は限られている。副作用の可能性はあるものの、臨床症状のコントロールにはしばしばグルココルチコイドのみが有効である。新たな治療法を検討する研究には、治療効果を評価するための皮膚病変のグレード評価に有効な客観的スコアリングシステムが必要である。

**仮説/目的:**

本研究の目的は、再発性蕁麻疹を有する馬の皮膚病変をグレード評価するための馬蕁麻疹活動性スコア(EqUAS)における観察者間および観察者内の信頼性を調査し、経験豊富な観察者と経験の浅い観察者の間の一致度を調べることであった。

**被験動物:**

治療臨床試験に登録された40頭のオーナー所有馬。

**材料と方法:**

各馬の体の両側10枚の標準化された写真を用いて、40の個別データセットを作成した。5人の盲検化された観察者がEqUASに従って写真を評定し、観察者間信頼性を算出した。観察者内信頼性は、2人の観察者がそれぞれ2回ずつ写真を採点して算出した。評点はピアソンの相関(r)で信頼性を、クラス内相関係数(ICC)で一致性を評価した。

**結果:**

観察者間信頼性はr=0.9036‐0.9600(p<0.0001)と良好であった。観察者内信頼性と一致度も、各観察者でr = 0.98(p<0.0001)、ICC = 0.945と優れていた。経験豊富な観察者と経験の浅い観察者の間の相関と一致も優れていた(r = 0.9615 [p < 0.0001])、ICC = 0.941)。

**結論と臨床的妥当性:**

EqUASは、慢性再発性蕁麻疹の馬の皮膚病変の評価において、優れた観察者内および観察者間の信頼性を示した。EqUASは臨床研究において有用なツールとなりうる。

## Resumo

**Contexto:**

A urticária recorrente é comum em cavalos. A fisiopatologia é pouco compreendida e as opções de tratamento são limitadas. Frequentemente, apenas glicocorticoides são eficazes no controle dos sinais clínicos, embora com potenciais efeitos adversos. Estudos que investigam novos tratamentos precisam de um sistema de escores objetivo validado para a classificação de lesões cutâneas e para avaliar a resposta.

**Hipótese/objetivos:**

Os objetivos foram investigar a confiabilidade inter e intraobservador do *Equine Urticaria Activity Score* (EqUAS) para a classificação de lesões cutâneas em cavalos com urticária recorrente e examinar a concordância entre observadores experientes e inexperientes.

**Animais:**

40 cavalos de propriedade privada inscritos em um ensaio clínico terapêutico.

**Materiais e Métodos:**

Dez fotografias padronizadas de ambos os lados do corpo de cada cavalo foram utilizadas para criar 40 bancos de dados individuais. Cinco observadores mascarados classificaram as fotografias de acordo com o EqUAS para o cálculo da confiabilidade interobservador. A confiabilidade intraobservador foi calculada a partir de dois observadores, cada um classificando as fotografias duas vezes. Os escores foram avaliados quanto à confiabilidade com a correlação de Pearson (r) e quanto à concordância com o coeficiente de correlação intraclasse (CCI).

**Resultados:**

A confiabilidade interobservador foi excelente, com r = 0,9036–0,9600 (p < 0,0001). A confiabilidade e a concordância intraobservador também foram excelentes, com r = 0,98 para cada observador (p < 0,0001) e CCI = 0,945. A correlação e a concordância entre observadores experientes e inexperientes foram excelentes (r = 0,9615 [p < 0,0001]) e CCI = 0,941).

**Conclusões e Relevância Clínica:**

O EqUAS apresentou excelente confiabilidade intra e interobservador para a avaliação de lesões cutâneas em cavalos com urticária crônica recorrente. Esta pode ser uma ferramenta útil em estudos clínicos.

## RESUMEN

**Introducción:**

La urticaria recurrente es común en caballos. Su fisiopatología es poco conocida y las opciones de tratamiento son limitadas. A menudo, solo los glucocorticoides son eficaces para controlar los signos clínicos, aunque con posibles efectos adversos. Los estudios que investigan nuevos tratamientos necesitan un sistema de puntuación objetivo validado para la clasificación de las lesiones cutáneas y así evaluar la respuesta.

**Hipótesis/objetivos:**

Los objetivos fueron investigar la fiabilidad interobservador e intraobservador del Equine Urticaria Activity Score (EqUAS) para la clasificación de las lesiones cutáneas en caballos con urticaria recurrente, y examinar la concordancia entre observadores experimentados e inexpertos.

**Animales:**

40 caballos de propiedad privada inscritos en un ensayo clínico terapéutico.

**Materiales y métodos:**

Se utilizaron diez fotografías estandarizadas de ambos lados del cuerpo de cada caballo para crear 40 conjuntos de datos individuales. Cinco observadores enmascarados clasificaron las fotografías según el EqUAS para calcular la fiabilidad interobservador. La fiabilidad intraobservador se calculó a partir de dos observadores que clasificaron las fotografías dos veces cada uno. Se evaluó la fiabilidad de las puntuaciones mediante la correlación de Pearson (r) y la concordancia con el coeficiente de correlación intraclase (ICC).

**Resultados:**

La fiabilidad interobservador fue excelente, con r = 0,9036–0,9600 (*p* < 0,0001). La fiabilidad y la concordancia intraobservador también fueron excelentes, con r = 0,98 para cada observador (*p* < 0,0001) y un ICC= 0,945. La correlación y la concordancia entre observadores experimentados e inexpertos fueron excelentes (r = 0,9615 [*p*< 0,0001] y un ICC= 0,941).

**Conclusiones y relevancia clínica:**

La EqUAS mostró una excelente fiabilidad intraobservador e interobservador para la evaluación de lesiones cutáneas en caballos con urticaria crónica recurrente. Puede ser una herramienta útil en estudios clínicos.

## INTRODUCTION

Chronic recurrent urticaria is a common clinical syndrome in horses. Horses appear to be highly susceptible to urticaria compared to other animals.^1^ Urticaria can be acute (<6–8 weeks duration) or chronic (>6–8 weeks duration). The pathogenesis of urticaria is still poorly understood. Immunologically mediated urticaria has been described as a Type I hypersensitivity to an allergen, with subsequent mast cell degranulation and release of histamine and other pro‐inflammatory mediators.^2^, ^3^ Possible inciting allergens for urticaria in horses range from insects to environmental allergens, foods, oral supplements, drugs and vaccines.^2^, ^4^, ^5^, ^6^ Type II and Type III hypersensitivities also have been described as mechanisms of urticaria.^7^ Current treatment options are limited. Systemic and topical treatment with corticosteroids, antihistamines, disinfectants and barrier‐repair products often are used, yet may have severe adverse effects or may lack sufficient efficacy.^1^, ^2^, ^8^ A prospective placebo‐controlled clinical trial is ongoing to evaluate a treatment for chronic urticaria in horses. To compare the severity of urticaria of enrolled horses, an Equine Urticaria Activity Score (EqUAS) was developed. In human medicine, the Urticaria Activity Score (UAS) is a commonly used diary‐based patient‐reported outcome measure that assesses itch severity and hive count in people with chronic spontaneous urticaria. The use of the UAS is recommended by international urticaria guidelines of several professional organisations.^9^ In dogs with atopic dermatitis (AD), several scoring systems such as the Canine Dermatitis Extent and Severity Index (CADESI) and the Canine Atopic Dermatitis Lesion Index (CADLI) have been developed to evaluate acute and chronic skin lesions. Several studies have shown that the CADESI is a valid and reliable scoring system for evaluation of disease severity and treatment success.^10^, ^11^ Several scoring systems have been developed for cats: Scoring Feline Allergic Dermatitis (SCORFAD) and the Feline Dermatitis Extent and Severity Index (FeDESI). These are used to assess allergic skin lesions and disease progression.^12^, ^13^ In horses, a reliable and validated clinical scoring system to evaluate disease progression and treatment success of chronic recurrent urticaria lesions has not been reported. The aim of our study was to assess the intra‐ and interobserver reliability of the recently developed EqUAS in horses, and the potential difference in EqUAS depending on the experience of the observer. We hypothesised that the EqUAS would be a reliable score for grading urticaria in horses regardless of the experience of the observer.

## MATERIALS AND METHODS

### Ethics

This study was conducted according to the standards of good scientific principles and Good Clinical Practice (GCP) guidelines. Written informed owner consent was obtained for the animals whose photographs were used in that study. All data used were obtained under an animal experiment licence approved by the animal experiment committees of all respective cantons in Switzerland (33608).

### Data source and study sample

**FIGURE 1:**
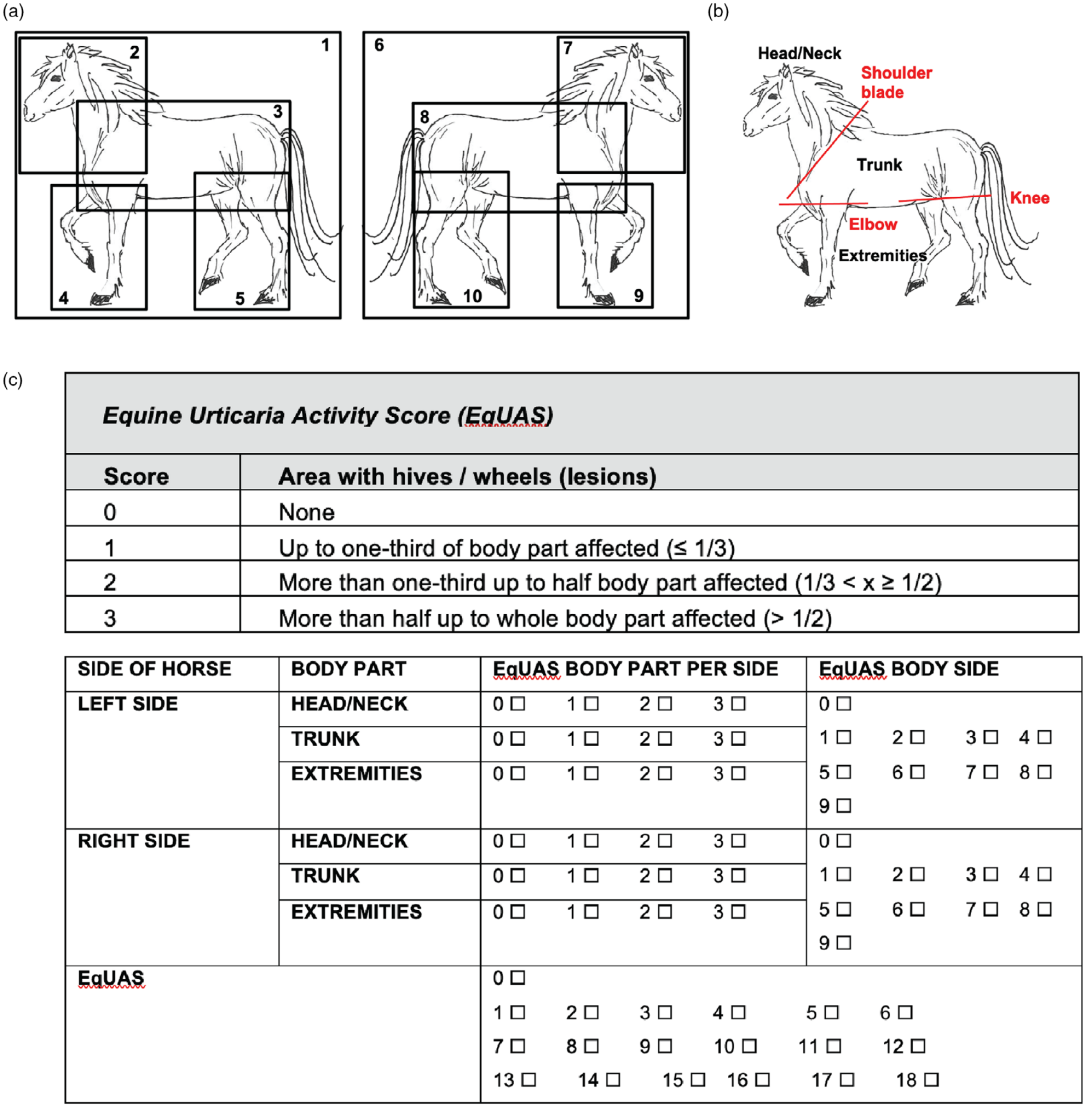
Description of the Equine Urticaria Activity Score (EqUAS). (a) Illustration of the photographic instructions for horse owners. (b) Illustration of the division of the right and left body sites into three different sections for regional scoring. (c) EqUAS for every body part and composition of the total EqUAS.

Forty client‐owned horses with chronic recurrent urticaria were enrolled. Ten photographs of each horse were used for this study. The datasets of photographs were collected as part of an efficacy and safety study for an active vaccination against interleukin (IL)‐5 for the treatment of chronic recurrent urticaria. The photographs were taken by respective owners according to a defined schedule (Figure 1a) on a weekly basis during the study period. All horses included in the study suffered from chronic recurrent urticaria, manifesting as chronic periodically reoccurring episodes of urticaria, with at least two or more urticaria episodes per year, starting ≥1 year before the study. All sexes and various breeds were included.

Datasets were randomly selected based on an even distribution of the severity of clinical signs: 10 datasets with a score of 0 (asymptomatic), 10 datasets with a score of 1–6 (low‐grade clinical signs), 10 datasets with a score of 7–12 (moderate‐grade clinical signs) and 10 datasets with a score of 13–18 (high‐grade clinical signs).

### Scoring system

The scoring system used (Figure 1c) was modified from the human UAS.^1^ It was adapted for horses to evaluate wheals only; pruritus was not assessed.

The EqUAS is composed of separate scores for the right and left body sides, which are further divided into three different body parts: (i) head/neck (up to and excluding the scapula), (ii) trunk (from and including the scapula up to and including the elbow/stifle) and (iii) extremities (from and excluding the elbow/stifle; medial aspect of right legs is scored from the left side, lateral aspect of right legs is scored from the right side, medial aspect of left legs is scored from the right side, lateral aspect of left legs is scored from the left side) (Figure 1b). For each body part and for each body side, an EqUAS Body Part Per Side score is given, ranging from 0 to 3, based on the following criteria: 0, for no wheals in the body part; 1, for up to one third of the body part affected (≤1/3); 2, for more than one third up to half the body part affected (1/3 < *x* ≤ 1/2); and 3, for more than half up to the whole body part affected (>1/2). The total EqUAS is the sum of all scores from the six body regions at a specific time point (Figure 1c) with a maximum score of 18 that can be obtained per horse and time point.

### Study design and observers

For the interobserver reliability, all datasets were graded in the same order by five observers within 1 week. Familiarity and expertise in the use of the EqUAS differed between the observers, who are all either veterinary surgeons or scientists with expertise in horses. Two of the five observers were familiar with the use of the EqUAS in live horses. One observer was experienced with dermatological diseases, especially urticaria, in horses and familiar with the use of the EqUAS from photographs. Two observers had no expertise with urticaria in horses or the use of the EqUAS. All observers were masked to the selection of datasets.

The scoring for the intraobserver reliability was performed by two experienced observers on two different days with ≥1 week between the two scoring time points. The order and labelling of the datasets were changed between the different time points.

### Statistical methods

All data were analysed using statistical software (prism, v10, GraphPad; R v4.4.1); *p* <0.05 were considered statistically significant.

For the interobserver reliability, pairwise correlation between the total scores per dataset from the five investigators was calculated using Pearson correlation. Agreement between the observers was examined by calculation of the intraclass correlation coefficient (ICC) with a 95% confidence interval (CI). The benchmarking of ICC values was adapted from previous studies:^14^ < 0.50: poor, 0.50–0.75: moderate, 0.76–0.90: good and >0.90: excellent reliability.

Intraobserver reliability was assessed by determining whether two measurements made by the same observer, on two distinct occasions, yielded similar results.^10^ The two scores given by the same observer for the same dataset were compared for reliability using Pearson correlation and agreement using ICC.

Agreement between experienced and inexperienced observers was calculated by ICC. Correlation of scoring between experienced and inexperienced observers was assessed by Pearson correlation with nonweighted least‐squares regression fit, computing side‐by‐side replicates and analysing means.

## RESULTS

### Interobserver reliability

**FIGURE 2:**
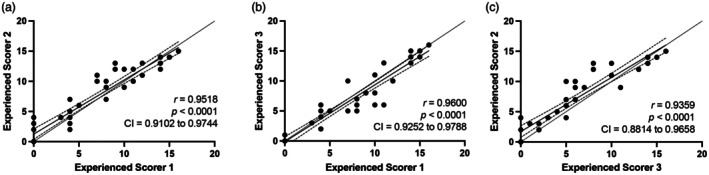
Evaluation of interobserver reliability of the Equine Urticaria Activity Score (EqUAS) between different observers. Pairwise Pearson correlation between (a) experienced scorer 1 and experienced scorer 2, (b) experienced scorer 1 and experienced scorer 3 and (c) experienced scorer 2 and experienced scorer 3. Complete results are available in Figure S1.

Results of the analyses of interobserver reliability of the EqUAS are provided in Figure 2 and Figure S1. Excellent interobserver reliability was found with the *r*‐values distributed between 0.9036 and 0.9600 (*p* < 0.0001). There was excellent agreement between observers (ICC = 0.919).

### Intraobserver reliability

**FIGURE 3:**
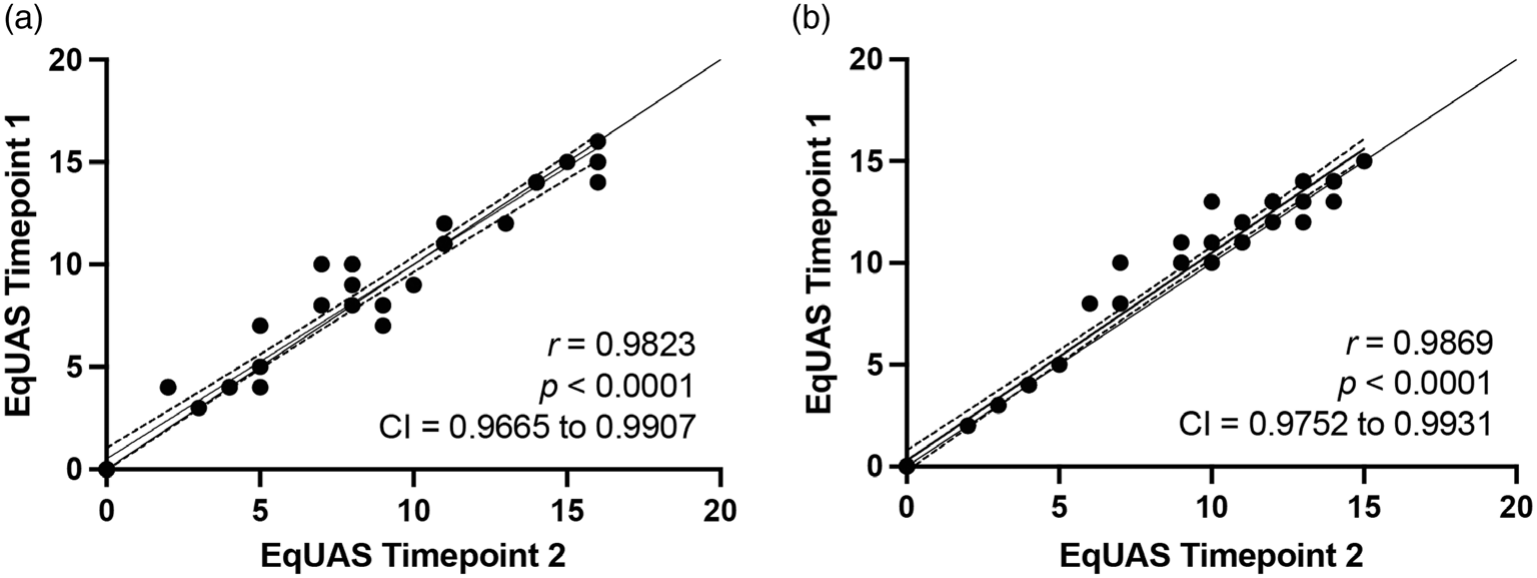
Evaluation of intraobserver reliability. Pearson correlation of the Equine Urticaria Activity Score (EqUAS) at two different time points of experienced scorer 1 (a) and experienced scorer 2 (b).

Results for intraobserver reliability are provided in Figure 3. Excellent intraobserver reliability was found (Observer 1: *r* = 0.9823; 95% CI 0.9665–0.9907; *p* < 0.0001; Observer 2: *r* = 0.9869; 95% CI 0.9752–0.9931, *p* < 0.0001). Agreement also was excellent (ICC = 0.945).

### Comparison of inexperienced to experienced observers

**FIGURE 4:**
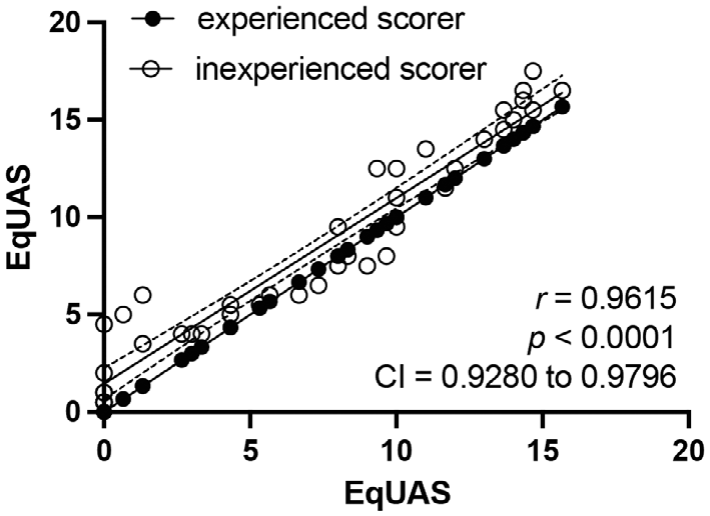
Agreement between experienced and inexperienced observers. Pearson correlation between experienced scorers (black filled circles) and inexperienced scorers (empty circles).

The correlation of scores between experienced and inexperienced observers was excellent (*r* = 0.9615; 95% CI 0.9280–0.9796; *p* < 0.0001) (Figure 4). There was a difference in mean total score for 36 of 40 datasets, ranging from 1 to 5 points, yet agreement was excellent (ICC = 0.941).

## DISCUSSION

The EqUAS showed excellent inter‐ and intraobserver reliability for grading of skin lesions in nonaffected and affected horses.

In humans, chronic spontaneous urticaria is a prevalent disease with a strong negative impact on patients' health‐related quality‐of‐life.^15^, ^16^ The results of our study are consistent with studies of the UAS used in people, where good levels of validity, reliability and sensitivity have been observed for the two versions of the score (UAS7 and UAS7_TD_).^15^, ^17^ The first validated scoring system in veterinary dermatology was the CADESI,^18^, ^19^ which is useful for comparing response to treatment. The fourth iteration of the CADESI is used in research and clinical settings with proven validity and reliability.^10^ In equine medicine, ordinal grading systems are used for the assessment of various diseases such as lameness, heart murmurs, ataxia and gastric ulceration. Moderate‐to‐substantial inter‐ and intraobserver reliability and agreement for these systems have been reported.^20^, ^21^, ^22^, ^23^ Given the widespread application of ordinal grading systems in veterinary clinical practice and research, determination of intra‐ and interobserver agreement and reliability of each system is important.^23^ Until now, no scoring system for dermatological lesions in horses with chronic recurrent urticaria has been validated. The EqUAS was developed based on the existing human and veterinary scoring systems to allow precise and objective evaluation of skin lesions in affected horses.

The excellent correlation and agreement between the EqUAS pairs validates the interobserver reliability of this score. Likewise, the nearly perfect correlation and lack of significant difference between the EqUAS values of the scoring show its excellent intraobserver reliability. Intraobserver reliability appears slightly better than interobserver reliability, with narrower 95% CI for intraobserver reliability and little overlap of CIs between inter‐ and intraobserver reliability. This possibly reflects the different interpretation of the EqUAS among individuals and the good ability of individual observers to repeatedly apply the grading system scale in the same way, as reported in other studies.^23^ Intraobserver reliability has been reported to be higher than interobserver reliability for other ordinal grading systems.^20^, ^23^, ^24^, ^25^ This may reflect consistency in the interpretation or application of the grading system within observers and slight differences in interpretation of the grading system among observers.^23^, ^26^

Differences in interpretation of a grading system have been speculated to be affected by clinical experience and opinions of the disorder being assessed.^25^ There was minimal influence of experience on the interobserver reliability of the EqUAS. Within the group of inexperienced observers, neither of the two inexperienced observers had previous experience with the clinical examination of horses with urticaria or the use of the EqUAS for scoring of urticaria lesions. The experienced observers were all specialists in equine medicine and/or had fundamental medical knowledge and extensive clinical and research experience in using the EqUAS. Our findings show that interobserver reliability of the EqUAS is not affected by familiarity with the grading system or by observer experience. A very mild influence of experience with the grading system was to be expected owing to the extended knowledge of experienced versus inexperienced personnel.

A limitation of our study was the fact that no power analysis could be performed to determine the adequate sample size, because there are no prevalence data available for chronic relapsing urticaria in horses. Another limitation was the use of photographs taken by the horse owners, rather than live assessment of lesions. Using photographs facilitated the conduction of the study, making standardisation and randomisation possible for all observers. It also facilitated the measurement of intraobserver reliability given that urticaria lesions can change very quickly. The quality of the photographs varied among the horses and the season, for example, as a consequence of heavy winter coat. Because all horses were only scored visually, small urticaria lesions, which cannot be observed yet probably could be palpated, were not taken into consideration.

The excellent inter‐ and intraobserver reliability of the EqUAS shown in this study makes this a useful scoring tool for remote examinations of horses with urticaria to observe disease progression. Further studies assessing the reliability of the EqUAS in clinical examinations of horses with urticaria should be conducted to confirm the excellent reliability.

## CONCLUSION

The EqUAS using photographs for grading urticaria lesions shows excellent intra‐ and interobserver reliability, and performs well regardless of clinicians' experience. The EqUAS is a valuable scoring system which can be used to evaluate urticaria in the clinical and scientific setting.

## AUTHOR CONTRIBUTIONS

**Katharina Birkmann:** Investigation; writing – review and editing; writing – original draft. **Nina Waldern:** Investigation. **Simone Jucker:** Investigation. **Katharina Balaschitsch:** Investigation. **Yury Zablotski:** Formal analysis. **Antonia Fettelschoss‐Gabriel:** Investigation; writing – review and editing.

## FUNDING INFORMATION

This project was supported by funding of the Swiss National Fond (SNF) BRIDGE to AF‐G and Evax AG.

## CONFLICT OF INTEREST STATEMENT

KBi and AF‐G are involved in the development of active immunotherapies. The authors NW, SJ, KBa and YZ have no conflicts of interest to disclose.

## Supporting information


Figure S1

## Data Availability

The data that supports the findings of this study are available in the supplementary material of this article.
