# Supplementary figures and images for: Validation of the Equine Urticaria Activity Score for the assessment of chronic recurrent urticaria in horses
Source: Vet Dermatol. 2025 May 19;36(5):630–7. doi: 10.1111/vde.13358 (PMC12420881; doi:10.1111/vde.13358)

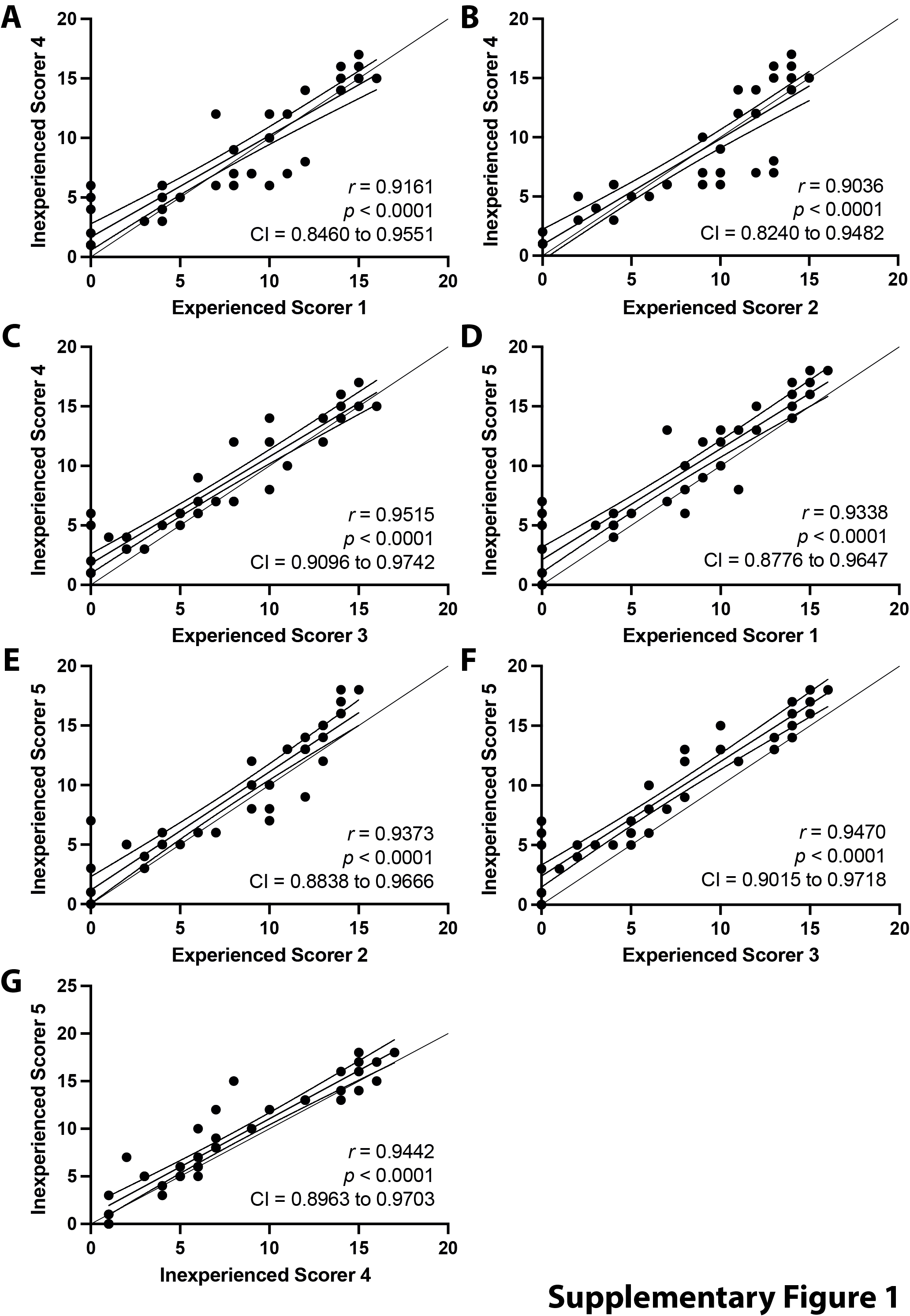

Supplement: Supplementary file 1 — Figure S1 [file VDE-36-630-s001.png]
